# Spiral search: a hydrophobic-core directed local search for simplified PSP on 3D FCC lattice

**DOI:** 10.1186/1471-2105-14-S2-S16

**Published:** 2013-01-21

**Authors:** Mahmood A Rashid, MA Hakim Newton, Md Tamjidul Hoque, Swakkhar Shatabda, Duc Nghia Pham, Abdul Sattar

**Affiliations:** 1 Institute for Integrated & Intelligent Systems, Griffith University, QLD, Australia; 2 Queensland Research Lab, National ICT Australia, QLD, Australia; 3 Computer Science, University of New Orleans, USA

## Abstract

**Background:**

Protein structure prediction is an important but unsolved problem in biological science. Predicted structures vary much with energy functions and structure-mapping spaces. In our simplified *ab initio *protein structure prediction methods, we use hydrophobic-polar (HP) energy model for structure evaluation, and 3-dimensional face-centred-cubic lattice for structure mapping. For HP energy model, developing a compact hydrophobic-core (H-core) is essential for the progress of the search. The H-core helps find a stable structure with the lowest possible free energy.

**Results:**

In order to build H-cores, we present a new Spiral Search algorithm based on tabu-guided local search. Our algorithm uses a novel H-core directed guidance heuristic that squeezes the structure around a dynamic hydrophobic-core centre. We applied random walks to break premature H-cores and thus to avoid early convergence. We also used a novel relay-restart technique to handle stagnation.

**Conclusions:**

We have tested our algorithms on a set of benchmark protein sequences. The experimental results show that our spiral search algorithm outperforms the state-of-the-art local search algorithms for simplified protein structure prediction. We also experimentally show the effectiveness of the relay-restart.

## Introduction

Proteins are essentially sequences of amino acids. They adopt specific folded three-dimensional structures to perform specific tasks. The function of a given protein is determined by its *native *structure, which has the lowest possible free energy level. Nevertheless, misfolded proteins cause many critical diseases such as Alzheimer's disease, Parkinson's disease, and Cancer [1,2]. Protein structures are important in drug design and biotechnology.

### PSP problem

Protein structure prediction (PSP) is computationally a very hard problem [3]. Given a protein's amino acid sequence, the problem is to find a three dimensional structure of the protein such that the total interaction energy amongst the amino acids in the sequence is minimised. The protein folding process that leads to such structures involves very complex molecular dynamics [4] and unknown energy factors. To deal with the complexity in a hierarchical way, researchers have used discretised lattice-based structures and simplified energy models [5-7] for PSP. However, the complexity of the simplified problem still remains challenging.

### The state-of-the-art approaches

There are a large number of existing search algorithms that attempt to solve the PSP problem by exploring feasible structures called *conformations*. A memory based local search (LS-Mem) [8] method reportedly produced the best results on face-centred-cubic (FCC) lattice for hydrophobic-polar (HP) energy model. Before LS-Mem, the state-of-the-art results were achieved for similar model by tabu-based local search (LS-Tabu) methods [9,10]. Besides these, genetic algorithms (GA) [11], and tabu search [12] found promising results on 2D and 3D hexagonal lattice based HP models.

### Research issues

In general, the success of single-point and/or population-based search algorithms crucially depends on the balance of diversification and intensification of the exploration. However, these algorithms often get stuck in local minima. As a result, they perform poorly on large sized (> 100 amino acids) proteins. Any further progress to these algorithms requires addressing the above issues appropriately.

### Our contributions

**Figure 1:**
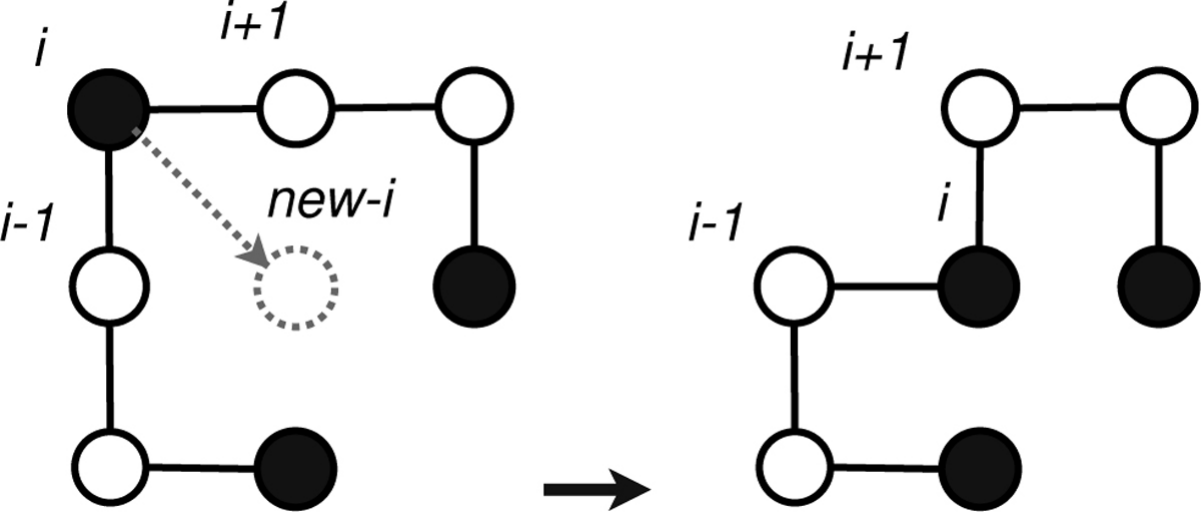
**Diagonal move**. Depiction of a diagonal move, for easy comprehension, shown in 2D space.

In this paper, we present a novel spiral search algorithm for *ab initio *protein structure prediction using HP energy model on three-dimensional (3D) FCC lattice. By using tabu heuristic, the search approaches towards the optimum solution by spinning around a dynamic hydrophobic-core centre (HCC) like a coil. We call our tabu-based spiral search algorithm **SS-Tabu**. In SS-Tabu, we consider the diagonal move (corner-flip) (as shown in Figure 1) to build the hydrophobic-core (H-core). We apply random-walk [13] to break the premature H-core. We use a novel relay-restart when the search is trapped in local minima and the random-walk fails to overcome the stagnation. On a set of benchmark proteins, SS-Tabu significantly outperforms the state-of-the-art local search algorithms [8,9] on similar models.

## Background

### Computational methods for PSP

*Homology modeling, protein threading *and *ab initio *are three computational approaches used in protein structure prediction. Prediction quality of *homology modeling *and *protein threading *depends on the sequential similarity of previously known protein structures. However, our work is based on the *ab initio *approach that only depends on the amino acid sequence of the target protein. Levinthal's paradox [14] and Anfensen's hypothesis [15] are the basis of *ab initio *method for protein structure prediction. The idea was originated in 1970 when it was demonstrated that all information needed to fold a protein resides in its amino acid sequence. In our simplified protein structure prediction model, we use 3D FCC lattice for conformation mapping, HP energy model for conformation evaluation, and a hydrophobic-core centric local search algorithm (SS-Tabu) for conformation search. Local search approach, 3D FCC lattice, and HP energy model are described below.

### Local search

Starting from an initial solution, local search algorithms move from one solution to another to find a better solution. Local search algorithms are well known for efficiently producing high quality solutions, which are difficult for systematic search approaches. However, they are incomplete [16], and suffer from revisitation and stagnation. Restarting the whole or parts of a solution remains the typical approach to deal with such situations. In PSP, Cebrian *et al. *[9] used a local search algorithm combined with tabu heuristic. They implemented their method on 3D FCC lattice configuration for HP model, and tested its effectiveness on Harvard instances [17]. Later, Dotu *et al. *[10] extended the work in [9] by using a hybrid method that combines local search and constraint programming together. Prior to LS-Mem, these two methods [9,10] produced the state-of-the-art results for PSP on FCC lattice and HP energy model.

### Tabu meta-heuristic

Tabu meta-heuristic [18,19] enhances the performance of local search algorithms. It maintains a memory structure to remember the local changes of a solution. Then any local changes for those stored positions are forbidden for certain number of subsequent iterations (known as tabu tenure).

### 3D FCC lattice

**Figure 2:**
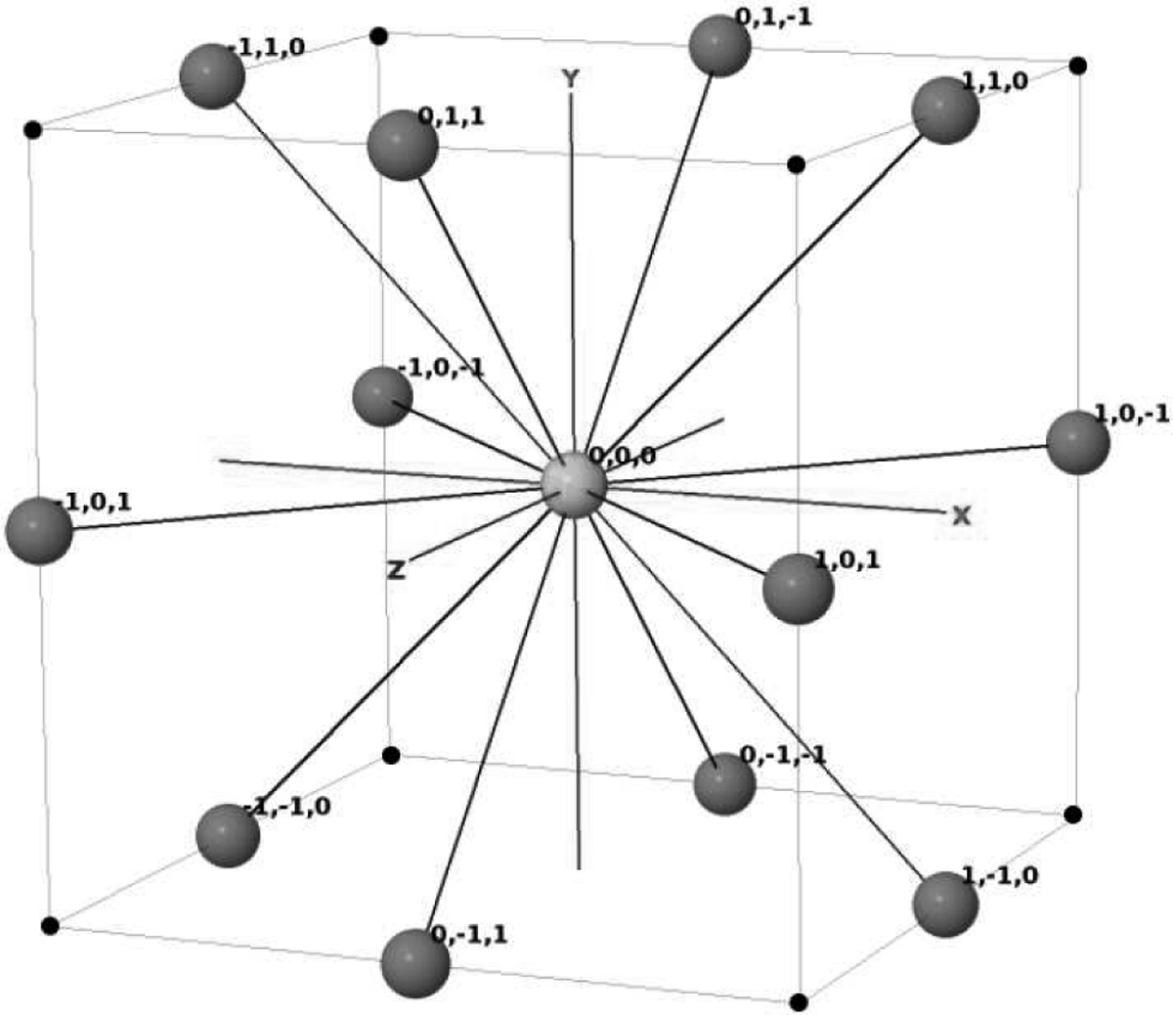
**3D FCC lattice**. A unit 3D FCC lattice; the 12 basis vectors are shown on the Cartesian coordinates; the vectors represent the 12 topological neighbours of the origin on the FCC lattice.

The FCC lattice has the highest packing density compared to the other existing lattices [20]. In FCC, each lattice point (the origin in Figure 2) has 12 neighbours with 12 *basis vectors *(1, 1, 0), (-1, -1, 0), (-1, 1, 0), (1, -1, 0), (0, 1, 1), (0, 1, -1), (1, 0, 1), (1, 0, -1), (0, -1, 1), (-1, 0, 1), (0, -1, -1), and (-1, 0, -1). The hexagonal closed pack (HCP) lattice, also known as cuboctahedron, was used in [11]. In HCP, each lattice point has 12 neighours that correspond to 12 basis vertices with real-numbered coordinates, which causes the loss of structural precision for PSP. In simplified PSP, conformations are mapped on the lattice by a sequence of basis vectors, or by the *relative vectors *that are relative to the previous basis vectors in the sequence.

### HP energy model

**Figure 3:**
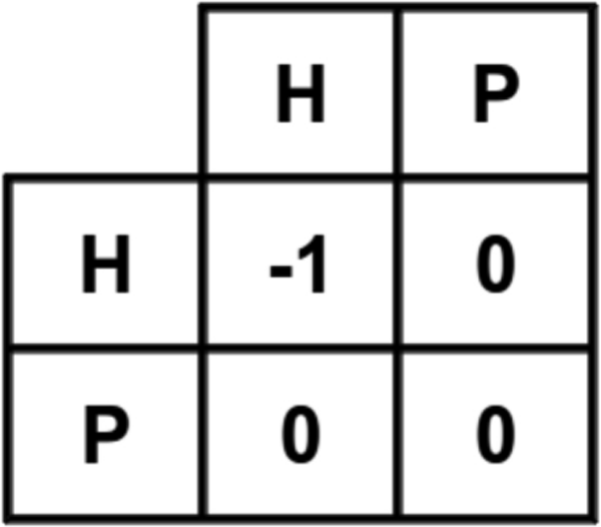
**HP model**. HP energy model [22].

The 20 constituent amino acids of proteins are broadly divided into two categories based on the hydrophobicity of the amino acids: (a) hydrophobic amino acids denoted as H (*Gly, Ala, Pro, Val, Leu, Ile, Met, Phe, Tyr, Trp*); and (b) hydrophilic or polar amino acids denoted as P (*Ser, Thr, Cys, Asn, Gln, Lys, His, Arg, Asp, Glu*). In the HP model [21,22], when two non-consecutive hydrophobic amino acids become topologically neighbours, they contribute a certain amount of negative energy, which for simplicity is shown as -1 in Figure 3. The total energy (*E*) of a conformation based on the HP model becomes the sum of the contributions of all pairs of non-consecutive hydrophobic amino acids as shown in Equation 1.

(1) $$E=\underset{i<j-1}{\sum }c_{ij}\cdot {e_{i}}_{j}$$

where *c_ij _*= 1 if amino acids *i *and *j *are non-consecutive neighbours on the lattice, otherwise 0; and *e_ij _*= -1 if *i_th _*and *j_th _*amino acids are hydrophobic, otherwise 0.

### Related work

Different types of metaheuristic have been used in solving the simplified PSP problem. These include Monte Carlo Simulation [23], Simulated Annealing [24], Genetic Algorithms (GA) [25,26], Tabu Search with GA [12], Tabu Search with Hill Climbing [27], Ant Colony Optimisation [28], Immune Algorithms [29], Tabu-based Stochastic Local Search [8,9], and Constraint Programming [10]. Cebrian *et al. *[9] used tabu-based local search, and Shatabda *et al. *[8] used memory-based local search with tabu heuristic and achieved the state-of-the-art results. However, Dotu *et al. *[10] used constraint programming and found promising results but only for small sized (< 100 amino acids) proteins. Besides local search, Unger and Moult [25] applied genetic algorithms to PSP and found their method to be more promising than the Monte Carlo based methods [23]. They used absolute encodings on the square and cubic lattices for HP energy model. Later, Patton [30] used relative encodings to represent conformations and a penalty method to enforce the self-avoiding walk constraint.

The GA has been used by Hoque *et al. *[11] for cubic, and 3D HCP lattices. They used Depth First Search (DFS) to generate pathways [31] in GA crossover for PSP. They also introduced a twin-removal operator [32] to remove duplicates from the population and thus to prevent the search from stalling.

## Methods

In HP model, protein structures have H-cores that hide the hydrophobic amino acids from water and expose the polar amino acids to the surface to be in contact with the surrounding water molecules [33]. H-core formation is the main objective of HP based PSP. To achieve this, the total distance of all H-H pairs is minimised in [9]. A predefined motif based segment replacement strategy is applied in [11] to replace structure segments by pre-determined substructures based on matching H-P orientations in the target sequence. In SS-Tabu, we try to reduce the distance of each H-amino acid from the HCC; which eventually helps minimise the free energy level of the conformation.

### Spiral search framework

**Figure 4:**
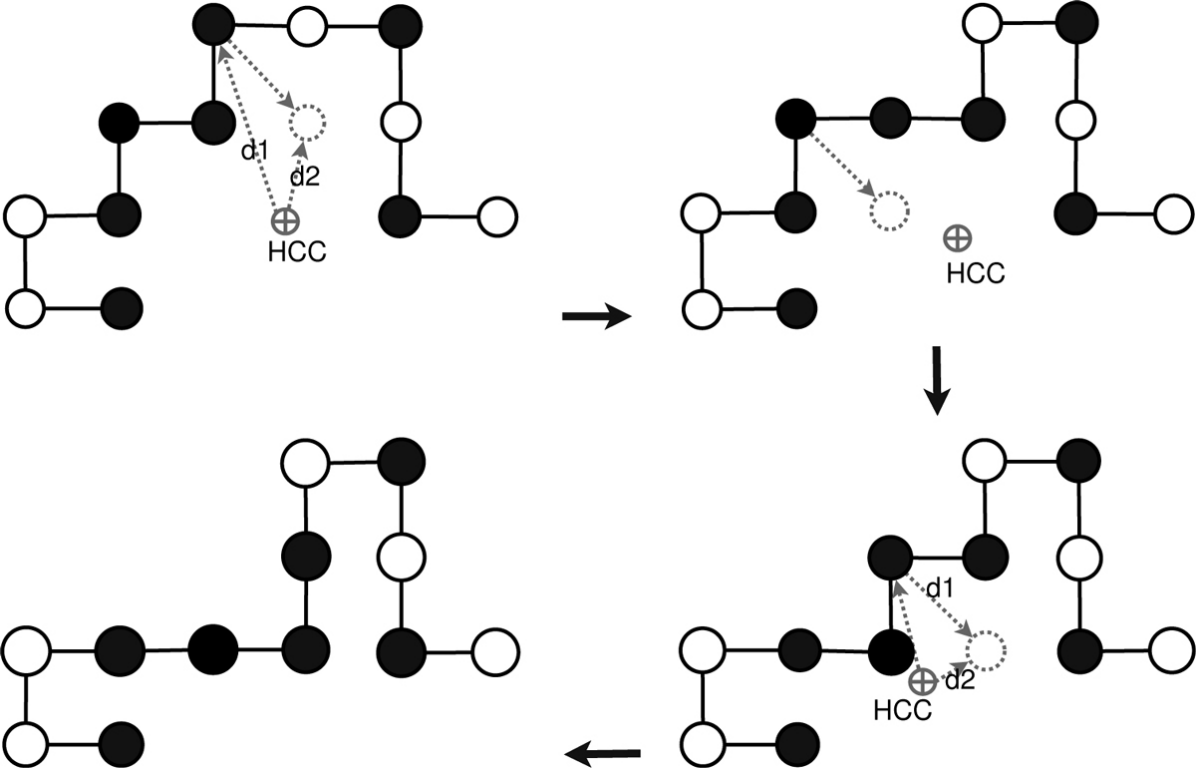
**Spiral search**. Spiral search comprising a series of diagonal moves. For simplification and easy understanding, the figures are presented in 2-dimensional space.

**Figure 5:**
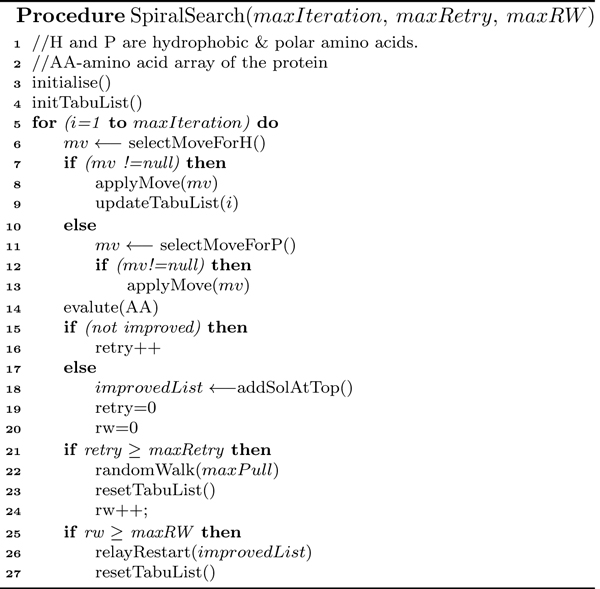
**Spiral search algorithm**. Spiral Search framework: *pseudocode *of Procedure *SpiralSearch*.

In spiral search, only the diagonal move operator is used repeatedly (as shown in Figure 4) in building H-cores. A diagonal move displaces *i*th amino acid from its position to another position on the lattice without changing the position of its succeeding (*i *+ 1)th and preceding (*i *- 1)th amino acids in the sequence. The move is just a corner-flip to an unoccupied lattice point. In SS-Tabu, we repeatedly use diagonal moves that squeeze the conformation and quickly form the H-core. The spiral search procedure (see the *pseudocode *in Figure 5) is composed of several sub-procedures mainly, for move selection, for handling local minima and stagnation, and for initialisation and evaluation.

#### Move selection

In move selection, the hydrophobic amino acids get priority in comparison to polar amino acids. The move selection criteria are explained in the following paragraphs.

##### H-move selection

**Figure 6:**
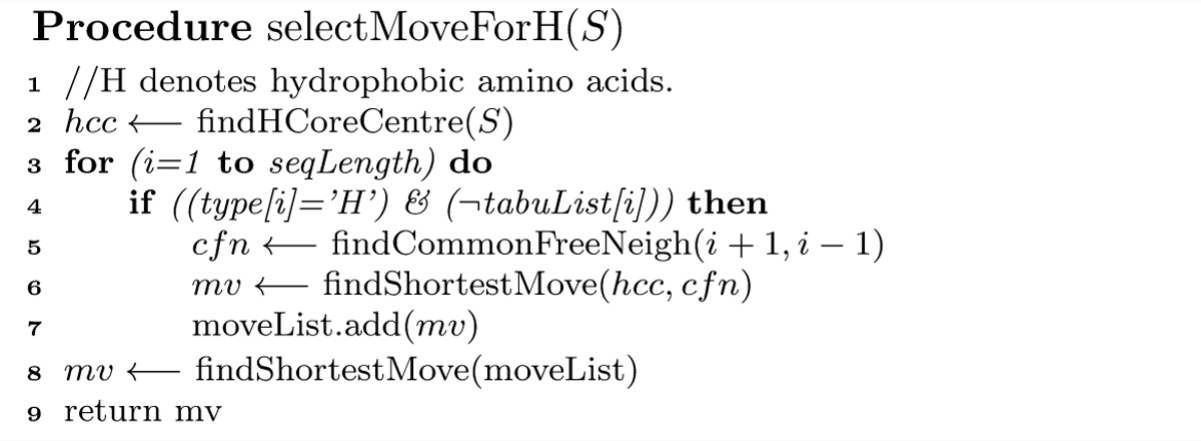
**H-move algorithm**. Spiral Search framework: *pseudocode *of Procedure *selectMoveForH*.

In H-move selection (see the *pseudocode *in Figure 6), the HCC is calculated by finding arithmetic means of *x*, *y*, and *z *coordinates of all hydrophobic amino acids using Equation 2. The selection is guided by the Cartesian distance *d_i _*(as shown in Equation 3) between HCC and the hydrophobic amino acids in the sequence. For the *i*th hydrophobic amino acid, the common topological neighbours of the (*i *- 1)th and (*i *+ 1)th amino acids are computed. The topological neighbours (TN) of a lattice point are the points at unit lattice-distance apart from it. For 3D FCC lattice, there are four common TN of the (*i *- 1)th and (*i *+ 1)th amino acids. From the common neighbours, the unoccupied points are identified. The Cartesian distance of all unoccupied common neighbours are calculated from the HCC using Equation 3. Then the point with the shortest distance is picked. This point is listed in the possible H-move list for *i*th hydrophobic amino acid if its current distance from HCC is greater than that of the selected point. When all hydrophobic amino acids are traversed and the feasible shortest distances are listed in H-move list, the amino acid having the shortest distance in H-move list is chosen to apply diagonal move operator on it. A tabu list is maintained for each hydrophobic amino acid to control the selection priority amongst them. For each successful move, the tabu list is updated for the respective amino acid. The process stops when no H-move is found. In this situation, the control is transferred to select and apply P-moves.

(2) $$x_{\text{hcc}}=\frac{1}{n_{\text{h}}}\overset{n_{\text{h}}}{\underset{i=1}{\sum }}x_{i},y_{\text{hcc}}=\frac{1}{n_{\text{h}}}\overset{n_{\text{h}}}{\underset{i=1}{\sum }}y_{i},\;z_{\text{hcc}}=\frac{1}{n_{\text{h}}}\overset{n_{\text{h}}}{\underset{i=1}{\sum }}z_{i}$$

where *n*_h _is the number of H amino acids in the protein.

(3) $$d_{i}=\sqrt{{\left(x_{i}-x_{\text{hcc}}\right)}^{2}+{\left(y_{i}-y_{\text{hcc}}\right)}^{2}+{\left(z_{i}-z_{\text{hcc}}\right)}^{2}}$$

##### P-move selection

For polar amino acids, the same kind of diagonal moves are applied as H-move. For each *i*th polar amino acid, all free lattice points that are common neighbours of lattice points occupied by (*i *- 1)th and (*i *+ 1)th amino acids are listed. From the list, a point is selected randomly to complete a diagonal move for the respective polar amino acid. No hydrophobic-core center is calculated, no Cartesian distance is measured, and no tabu list is maintained for P-move. After one try for each polar amino acid the control is returned to select and apply H-moves.

#### Stagnation recovery

For hard optimisation problems such as protein structure prediction, local search algorithms often face stagnation. Thus, handling such situation intelligently is important to proceed further. In our SS-Tabu, we use random-walk [13] and a new relay-restart technique with on-demand basis to deal with stagnation.

##### Random-walk

**Figure 7:**
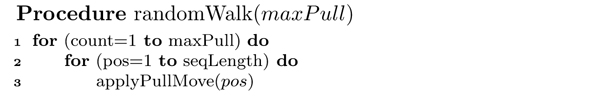
**Random-walk algorithm**. Spiral Search framework: *pseudocode *of Procedure *randomWalk*.

**Figure 8:**
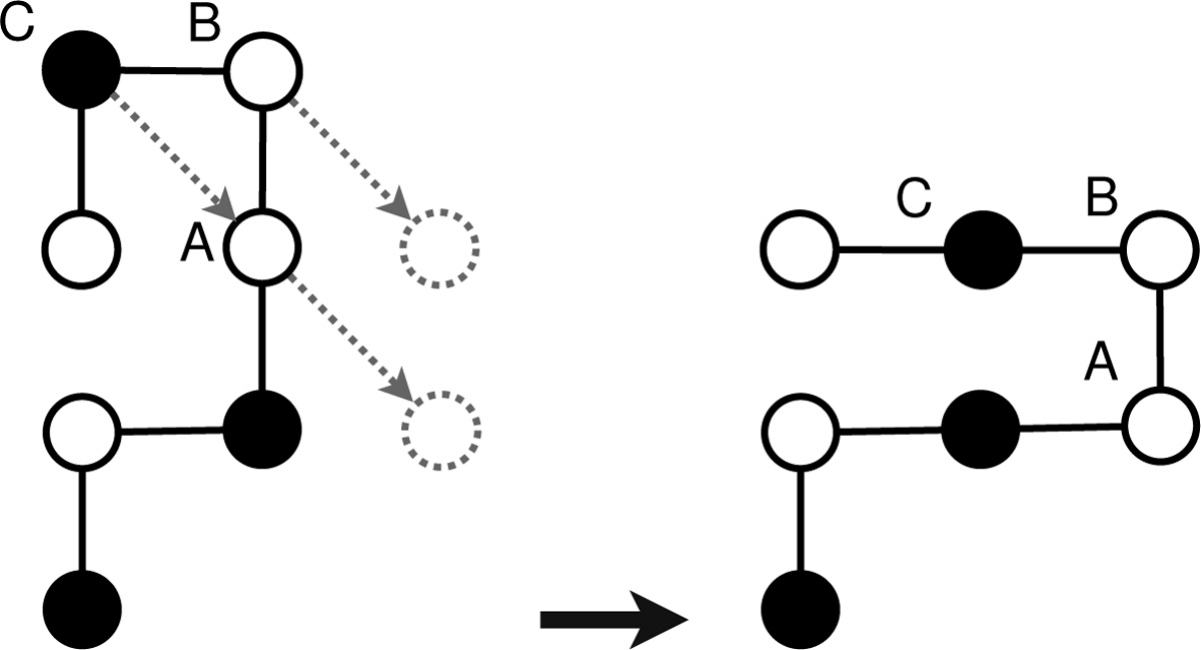
**Pull move**. The pull move operator [34] used in random-walk for collapsing a premature H-core; for easy comprehension, presented in 2D space.

Premature H-cores are observed at local minima. To escape local minima, a random-walk [13] algorithm (see the *pseudocode *in Figure 7) is applied. This algorithm uses pull moves [34] (as shown in Figure 8) to break the H-core. We use pull-moves because they are complete, local, and reversible. Successful pull moves never generate infeasible conformations. During pulling, energy level and structural diversification are observed to maintain a balance among these two. We allow energy level to change within 5% to 10% with changes in the structure from 10% to 75% of the original. We try to accept the conformation that is close to the current conformation in terms of energy level but is diverse in terms of structure.

##### Relay-restart

**Figure 9:**
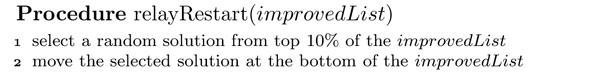
**Relay-restart algorithm**. Spiral Search framework: *pseudocode *of Procedure *relayRestart*.

Instead of using a fresh restart or restarting from the current best solution [8,9], we use a new relay-restart technique (see the *pseudocode *in Figure 9) when the search stagnation situation arises. We use relay-restart when random-walk fails to escape from local minima. The relay restart starts from an improving solution. We maintain an improving solution list that contains all the improving solutions after initialisation. When a solution with energy level better than the current global best is found, the solution is added to the top of the list pushing existing solutions back. For relay-restart, a random conformation from the top 10% solutions of the list is selected to start with. The selected solution is then sent back to the bottom of the list to keep it away from the scope of reselection in very near future.

#### Further implementation details

Like other local search algorithms, our spiral search requires initialisation. It also needs evaluation of the solution in each iteration. Further, it needs to maintain a tabu meta-heuristic to guide the search.

##### Initialisation

**Figure 10:**
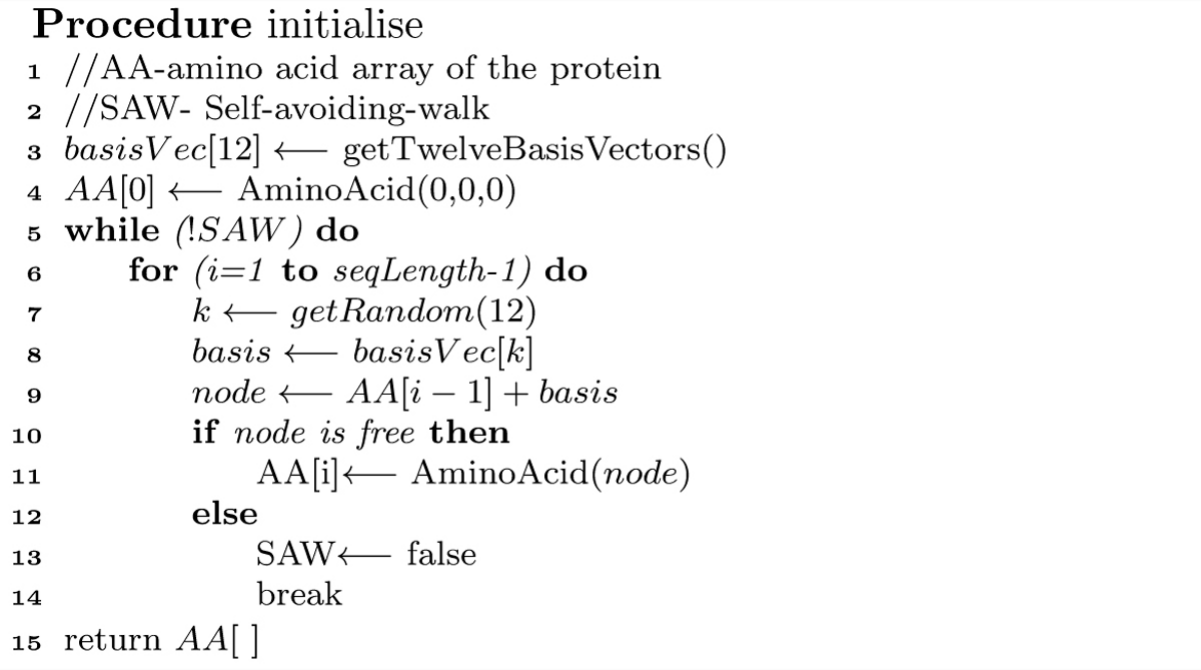
**Initialisation algorithm**. Spiral Search framework: *pseudocode *of Procedure *initialise*.

Our algorithm starts with a feasible conformation. We generate an initial conformation following a self-avoiding walk (SAW) on FCC lattice points. The *pseudocode *of the algorithm is presented in Figure 10. It places the first amino acid at (0, 0, 0). It then randomly selects a basis vector to place the successive amino acid at a neighbouring free lattice point. The mapping proceeds until a self-avoiding walk is found for the whole protein sequence.

##### Tabu tenure

Intuitively we use different tabu-tenures based on the number of hydrophobic amino acids (hCount) in the sequence. We intuitively calculate tabu-tenure using the formula in Equation 4:

(4) $$\text{tenure}=\left(10+\frac{\text{hCount}}{10}\right)$$

##### Evaluation

**Figure 11:**
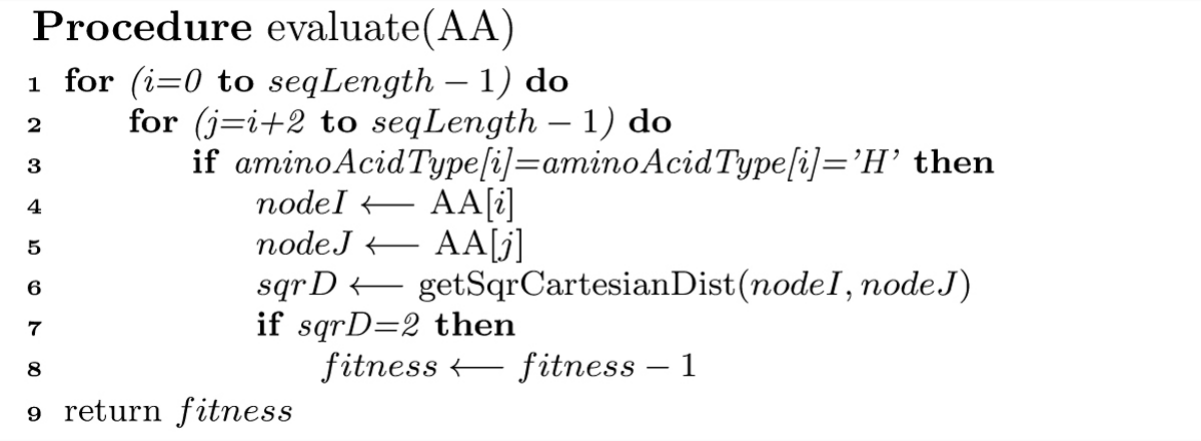
**Evaluation algorithm**. Spiral Search framework: *pseudocode *of Procedure *evaluate*.

After each iteration, the conformation is evaluated by counting the H-H contacts (topological neighbour) where the two amino acids are non-consecutive. The *pseudocode *in Figure 11 presents the algorithm of calculating the free energy of a given conformation. Note that energy value is negation of the H-H contact count.

## Results and discussion

In our experiment, the protein instances (as shown in Table 1), F180 and R instances are taken from Peter Clote laboratory website (*bioinformatics.bc.edu/clotelab/FCCproteinStructure*). Cebrian *et al. *[9], Dotu *et al. *[10], and Shatabda *et al. *[8] used these instances in evaluating their algorithms. We also use six more larger sequences that are taken from the CASP (*predictioncenter.org/casp9/targetlist.cgi*) competition. The corresponding CASP target IDs for proteins *3mse*, *3mr7*, *3mqz*, *3no6*, *3no3*, and *3on7 *are *T0521*, *T0520*, *T0525*, *T0516*, *T0570*, and *T0563*. These CASP targets are also used in [8]. To fit in the HP model, the CASP targets are converted to HP sequences based on the hydrophobic properties of the constituent amino acids. The lower bounds of the free energy values (in Column *LB*-*FreeE *of Table 1) are obtained from [8,9]; however, there are some unknown values (presented as ?) of lower bounds of free energy for large sequences.

**Table 1:** Experimental results of LS-Mem, LS-Tabu, and SS-Tabu

			The state-of-the-art				Spiral Search		Time/run
				
**Protein Info**.			LS-Mem		LS-Tabu		SS-Tabu		
**ID**	**Size**	**LB-FreeE**	**Best**	**Avg**	**Best**	**Avg**	**Best**	**Avg**	**min**

F90_1		-168			-164	-160	=	**-166**	
F90_2		-168			-165	-158	=	**-164**	
F90_3	90	-167	n/a	n/a	-165	-159	=	-**165**	120
F90_4		-168			-165	-159	=	**-165**	
F90_5		-167			-165	-159	=	**-165**	

S1	135	-357			-351	-341	**-355**	**-347**	
S2	151	-360	n/a	n/a	**-355**	-343	-354	**-347**	120
S3	162	-367			-355	-340	**-359**	**-350**	
S4	164	-370			-354	-343	**-358**	**-350**	

F180_1		-378	**-360**	-334	-338	-327	-357	**-340**	
F180_2	180	-381	**-362**	-340	-345	-334	-359	**-345**	300
F180_3		-378	-357	-343	-352	-339	**-362**	**-353**	

R1		-384	-353	-326	-332	-318	**-359**	**-345**	
R2	200	-383	-351	-330	-337	-324	**-358**	**-346**	300
R3		-385	-352	-330	-339	-323	**-365**	**-345**	

3mse	179	-323	-278	-254	-266	-249	**-289**	**-280**	
3mr7	189	-355	-311	-292	-301	-287	**-328**	**-313**	
3mqz	215	-474	-415	-386	-401	-383	**-420**	**-403**	300
3no6	229	-455	-400	-375	-390	-373	**-411**	**-391**	
3no3	258	-494	-397	-361	-388	-359	**-412**	**-393**	
3on7	279	?	-499	-463	-491	-461	**-512**	**-485**	

Results for 21 small (size < 100), medium (100 < size < 200), and large (size ≥ 200) sized proteins. The sequences are taken from [8-10] (S, F and R instances) and CASP competition (others). The results are calculated over 50 different runs with identical settings for each algorithm; duration of each run is presented in last column. The **bold-faced **values are the winners in respective rows. Column *LB*-*FreeE *presents the lower bounds of free energy.

Note: = denotes the lower bound of free energy is found and ? denotes unknown.

In Table 1, the *Size *column presents the number of amino acids in the sequences, and *LB-FreeE *column shows the known lower bounds of free energy for the corresponding protein sequences in Column *ID*. However, lower bound of free energy for protein *3on7 *is unknown. The best and average free energy for three different algorithms are also present in the table. The **bold-faced **values indicate better performance in comparison to the other algorithms for corresponding proteins. The experimental results show that our SS-Tabu wins over LS-Mem and LS-Tabu over the 21 proteins with a significant margin on average search results.

### 

#### Relative improvement

The difficulty to improve energy level is increased as the predicted energy level approaches to the lower bound. For example, if the lower bound of free energy of a protein is -100, the efforts to improve energy level from -80 to -85 is much less than that to improve energy level from -95 to -100 though the change in energy is the same (-5). Relative Improvement (RI) explains how close our predicted results to the lower bound of free energy with respect to the energy obtained from the state-of-the-art approaches.

In Table 2, we present a comparison of improvements (%) on average conformation quality (in terms of free energy levels). We compare SS-Tabu (target) with LS-Tabu and LS-Mem (references). For each protein, the RI of the target (*t*) w.r.t. the reference (*r*) is calculated using the formula in Equation 5, where *E_t _*and *E_r _*denote the average energy values achieved by target and reference respectively, and *E*_1 _is the lower bound of free energy for the protein in the HP model. We present the relative improvements only for the proteins having known lower bound of free energy values. We test our new algorithm on 21 different proteins of various length. The **bold-faced **values are the minimum and the maximum improvements for the same column.

**Table 2:** Relative improvement by SS-Tabu w.r.t. LS-Mem and LS-Tabu

			Target(t)	Reference(r)			
			
Protein Info.			SS-Tabu	LS-Mem		LS-Tabu	
**ID**	**Size**	**LB-FreeE**	**Avg (*E_t_*)**	**Avg (*E_r_*)**	**RI**	**Avg (*Er*)**	**RI**

F90_1		-168	-166			-160	**75.00%**
F90_2		-168	-164			-158	60.00%
F90_3	90	-167	-165	n/a	n/a	-159	**75.00%**
F90_2		-168	-165			-159	66.67%
F90_3		-167	-165			-159	**75.00%**

S1		-357	-347			-341	37.50%
S2	200	-360	-347	n/a	n/a	-343	23.52%
S3		-367	-350			-340	37.03%
S4		-370	-350			-343	25.92%

F180_1		-378	-340	-334	13.64%	-327	25.49%
F180_2	180	-381	-345	-340	**12.20%**	-334	23.40%
F180_3		-378	-353	-343	28.57%	-339	35.90%

R1		-384	-345	-326	32.76%	-318	40.91%
R2	200	-383	-346	-330	30.19%	-324	37.29%
R3		-385	-345	-330	27.27%	-323	35.48%

3mse	179	-323	-280	-254	**37.68%**	-249	41.89%
3mr7	189	-355	-313	-292	33.33%	-287	38.24%
3mqz	215	-474	-403	-386	19.32%	-383	**21.98%**
3no6	229	-455	-391	-375	20.00%	-373	21.95%
3no3	258	-494	-393	-361	24.06%	-359	25.19%
3on7	279	?	-485	-463	..	-461	..

The relative improvements of SS-Tabu w.r.t. LS-Mem and LS-Tabu are presented in the table. The column *RI *presents the relative improvements. The values are calculated using the formula in Equation 5. The value ? denotes an unknown value. The results are calculated over 50 different runs with identical settings for each algorithm. The **bold-faced **values are the minimum and maximum values of relative improvements for the respective columns. The column *LB-FreeE *presents the lower bounds of free energy.

Note: ? denotes unknown.

(5) $$\text{RI}=\frac{E_{t}-E_{r}}{E_{1}-E_{r}}*100\%$$

##### Improvement w.r.t. LS-Mem

The experimental results in Table 2, at column *RI *(relative improvement) under LS-Mem shows that our SS-Tabu is able to improve the search quality in terms of minimizing the free energy level over all 21 proteins. The relative improvements with respect to LS-Mem range from 12.20% to 37.68%.

##### Improvement w.r.t. LS-Tabu

The experimental results in Table 2, at column *RI *under LS-Tabu shows that our SS-Tabu is able to improve the search quality in terms of minimising the free energy level over all 21 proteins. The relative improvements with respect to LS-Tabu range from 21.95% to 75.00%.

##### Effectiveness of relay-restart

In Table 3, we present another set of experimental results to show the effectiveness of relay-restart in the spiral search framework. The results under the headings *Target *and *Reference *are obtained by running SS-Tabu respectively with and without relay-restart. The relative improvements on average search results are presented in the last column of the table. The relative improvements after including relay-restart in our SS-Tabu, are as minimum as 1.39% and as maximum as 23.08%.

**Table 3:** Effectiveness of relay-restart in SS-Tabu

**Protein Info**.			Reference(r)		Target(t)		RI (on avg using RR)
				
			SS-Tabu without RR		SS-Tabu with RR		
	
ID	Size	LB-FreeE	Best	Avg	Best	Avg	
F180_1		-378	-355	-333	-357	**-340**	15.56%
F180_2	180	-381	-358	-338	-359	**-345**	16.28%
F180_3		-378	-365	-346	-362	**-353**	21.88%

R1		-384	-362	-336	-359	**-345**	18.75%
R2	200	-383	-362	-340	-358	**-346**	13.95%
R3		-385	-362	-333	-365	**-345**	**23.08%**

3mse	179	-323	-291	-275	-289	**-280**	10.42%
3mr7	189	-355	-323	-309	-328	**-313**	8.70%
3mqz	215	-474	-423	-402	-420	**-403**	**1.39%**
3no6	229	-455	-412	-389	-411	**-391**	3.03%
3no3	258	-494	-423	-386	-412	**-393**	6.48%
3on7	279	?	-510	-484	-512	**-485**	..

Comparison between two different variants of SS-Tabu: *i*) The results under the heading Reference are obtained by running SS-Tabu without relay-restart (RR) and *ii*) the results under the heading *Target *are obtained by running SS-Tabu with relay-restart technique. The results are calculated over 50 different runs with identical settings for each algorithm. The **bold-faced **values in Columns *Avg *are the winners for the respective proteins, and that in Column *RI *are the minimum and maximum values of relative improvements. Column *LB-FreeE *presents the lower bounds of free energy.

Note: ? denotes unknown.

##### Simplified structure

**Figure 12:**
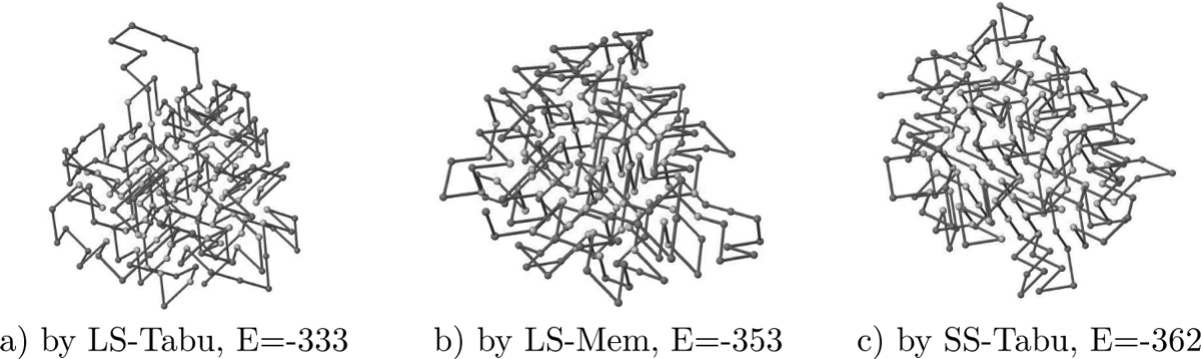
**Structure comparison**. The 3D structures of protein *R1 *obtained by a) LS-Tabu, b) LS-Mem, and c) SS-Tabu.

In Figure 12, we show the best structures found by SS-Tabu, LS-Mem and LS-Tabu for protein *R*1. Each algorithm runs over a period of 5 hours to achieve the results.

#### Search progress

**Figure 13:**
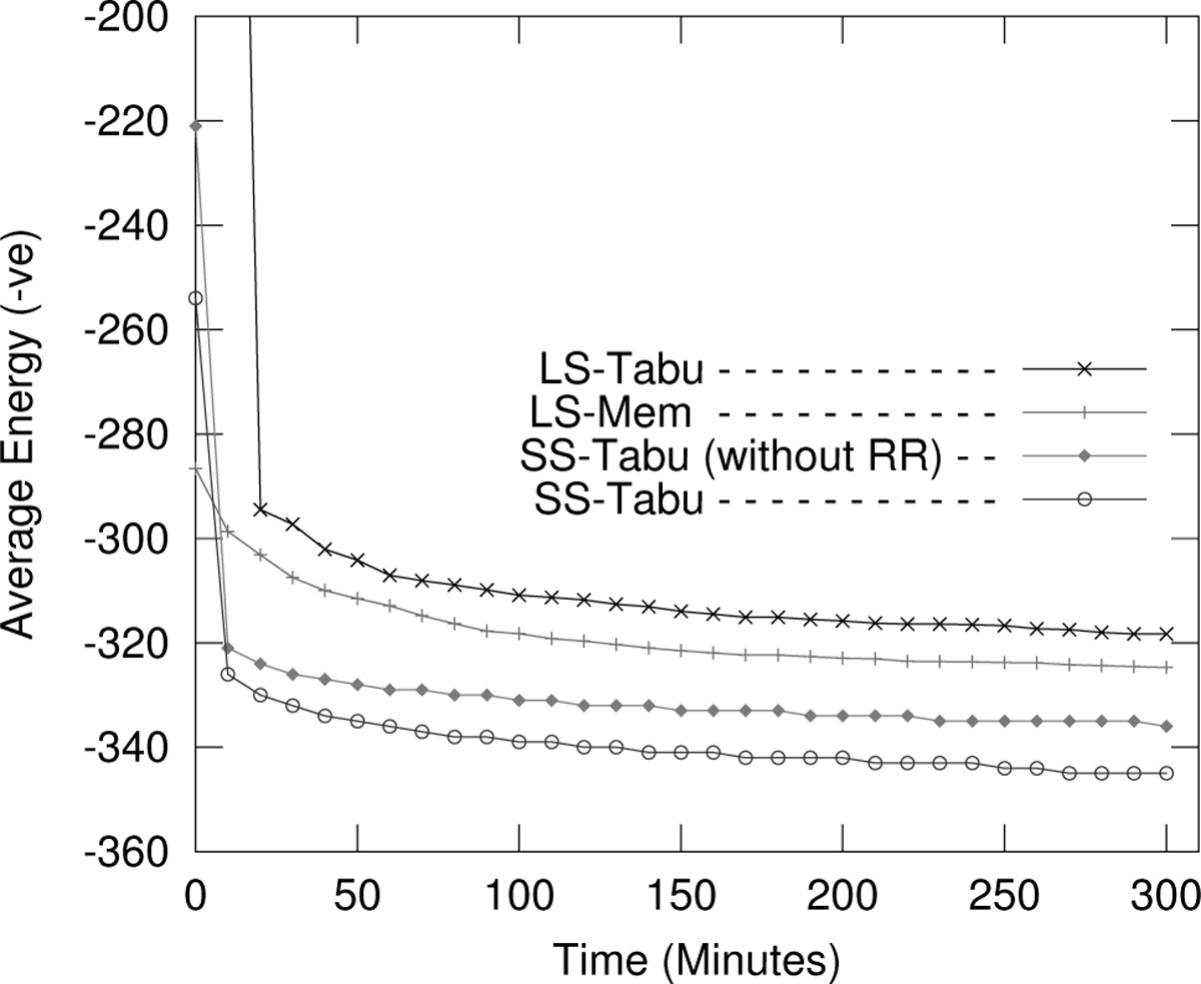
**Search progress**. Search progress for protein *R1 *with time for different approaches. The results are calculated over 50 different runs with identical settings for each algorithm.

We compare the search progresses of different variants of local search; LS-Tabu, LS-Mem, and SS-Tabu over time. Figure 13 shows the average energy values obtained with times by the algorithms for protein R1. We observe that all of the algorithms achieve very good progress initially, but with time increasing, our spiral search **SS-Tabu **makes more progress than LS-Tabu and LS-Mem.

## Conclusion

In this paper, we present a new spiral search (SS-Tabu) under the local search framework for simplified protein structure prediction on 3D face-centred-cubic lattice. We use a new search guiding heuristic, which is the distance of a hydrophobic amino acid from a dynamic hydrophobic-core centre. We also use a novel relay-restart technique to break the stagnation. We compare our results with two other local search algorithms: LS-Tabu and LS-Mem, which achieved the state-of-the-art results for similar models. We found that our SS-Tabu significantly outperforms both LS-Mem and LS-Tabu. We aim to apply our algorithm in high resolution protein structure prediction in future.

## Competing interests

The authors declare that they have no competing interests.

## Authors' contributions

MAR conceived the idea of *Spiral Search *algorithm. MAHN, MTH, SS, DNP, and AS helped MAR modeling, implementing, and testing the algorithm. All authors equally participated in analysing the test results to improve the algorithm and were significantly involved in the process of writing and reviewing the manuscript. SS also provided experimental data from his memory based local search (LS-Mem).

## Declarations

NICTA, the sponsor of the article for publication, is funded by the Australian Government as represented by the Department of Broadband, Communications and the Digital Economy and the Australian Research Council through the ICT Centre of Excellence program.

This article has been published as part of *BMC Bioinformatics *Volume 14 Supplement 2, 2013: Selected articles from the Eleventh Asia Pacific Bioinformatics Conference (APBC 2013): Bioinformatics. The full contents of the supplement are available online at http://www.biomedcentral.com/bmcbioinformatics/supplements/14/S2.
